# Intestinal Microbiome Metabolism of Cranberry (*Vaccinium macrocarpon*) Proanthocyanidin Dimers, but Not Trimers, Is Altered by Dysbiosis in Ulcerative Colitis *Ex Vivo*

**DOI:** 10.1021/acs.jafc.4c00042

**Published:** 2024-02-13

**Authors:** Maritza S. Diaz, Susanne U. Mertens-Talcott, Stephen T. Talcott

**Affiliations:** Department of Food Science and Technology, Texas A&M University, 370 Olsen Blvd., College Station, Texas 77845-2254, United States

**Keywords:** proanthocyanidins, cranberry, Vaccinium macrocarpon, ulcerative colitis, microbiome, fecal fermentation, polyphenols, tannins

## Abstract

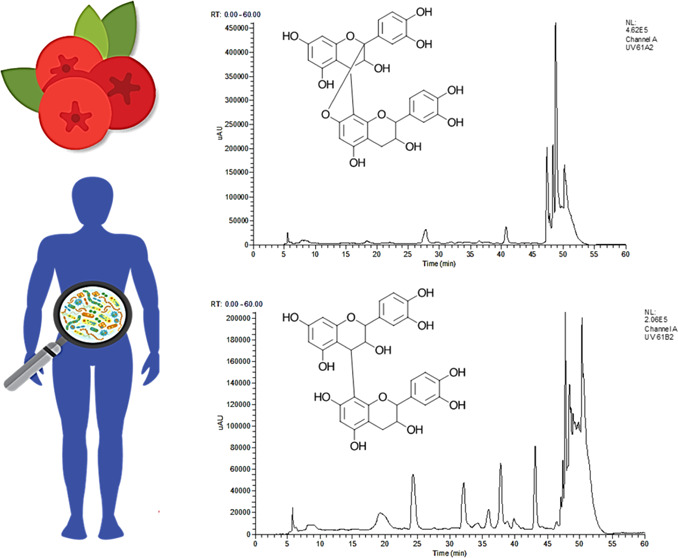

Cranberries contain proanthocyanidins with different interflavan bond types and degrees of polymerization. These chemical differences may impact the metabolism of proanthocyanidins by the intestinal microbiome. In our previous study, we found that healthy microbiomes produced higher concentrations of the phenolic acid metabolites 5-(3′,4′-dihydroxyphenyl)-*g*-valerolactone and 3-hydroxyphenylacetic acid from the cranberry extract in comparison to ulcerative colitis (UC) microbiomes *ex vivo*. To understand this difference, LC-ESI-MS/MS was utilized to characterize the metabolism of the precursor proanthocyanidins. Healthy microbiomes metabolized procyanidin A2, procyanidin B2, and procyanidin dimeric intermediates but not A-type trimers, to a greater extent than UC microbiomes. The metabolism of procyanidin A2 and procyanidin B2 by fecal microorganisms was then compared to identify their derived phenolic acid metabolites. 5-(3′,4′-Dihydroxyphenyl)-*g*-valerolactone and 3-hydroxyphenylacetic acid were identified as unique metabolites of procyanidin B2. Based on these results, the metabolism of procyanidin B2 contributed to the differential metabolism observed between healthy and UC microbiomes.

## Introduction

Proanthocyanidins (PACs), also known as condensed tannins, are a ubiquitous class of polyphenols that are found in many global food commodities. Their estimated dietary consumption is 0.1–0.5 g per day from common dietary sources including apples, cranberries, cacao, peanuts, cinnamon, avocados, and wine.^1^ Despite the abundance of PACs in the diet, the metabolism and physiological effects of PACs are still poorly understood. The complexity of their metabolic fate is due to interindividual differences in metabolism caused by the health status, the gut microbiome makeup, and the structural diversity of PACs.^2,3^

PACs range in chemical diversity by the degree of polymerization (DP), type of interflavan bonds between monomers, and identity of the monomers. PACs specifically are polymers of the flavonoid diastereomers, flavan-3-ols, and are formed through the condensation of flavan-3-ols through interflavan bonds, typically between C4 and C8 of two unique flavan-3-ol units and less commonly between C4 and C6.^1^ PACs with one interflavan bond between monomers are referred to as B-type and are the most predominant type of PAC found in fruits and botanicals. By contrast, foods such as cranberry, peanuts, avocado, and cinnamon contain the less common A-type PACs, which are differentiated by a second ether bond between C2 and C7 of two flavan-3-ol monomers. A-type PACs may contain one or more A-type linkages, in addition to B-type linkages, within the same polymer.^4^ For these reasons, there is a diverse range of PACs that exist in nature.

Cranberries (*Vaccinium macrocarpon*) contain a diverse range of PACs, which is hypothesized to be a reason for their high bioactivity. Notably, cranberries contain both A- and B-type PACs that can have a DP of up to 38.^5^ Through thiolysis reactions of cranberry PACs, the primary monomer of cranberry PACs has been identified as (−)-epicatechin.^4^ However, A-type, B-type, and different DP PACs have been found to have different physiological activities. Specifically, A-type PACs from cranberries are speculated to mitigate urinary tract infections through antiadhesion mechanisms against *p*-fimbriated, adherent-invasive *Escherichia coli*.^6^ However, due to their high molecular weight, it is widely debated whether PACs above a DP of 2 have sufficient bioavailability to exhibit efficacy in the urinary tract.^7^ Due to their low absorption in the small intestine and subsequent localization in the large intestine, recently, more focus has been put on the role of PACs and their biotransformation in the gastrointestinal system.^3^ It has been found that PACs may promote intestinal health by producing absorbable and bioactive metabolites, stimulating the growth of symbionts, promoting the production of mucins, and reducing intestinal inflammation.^8^ For these reasons, it is important to understand how different interflavan bond types and DP impact the metabolic fate of cranberry PACs.

Static fermentation models have been utilized to elucidate metabolites that are derived from A-type, B-type, and different DP PACs by the microbiome that could be responsible for conferring health benefits. Identified metabolites of PACs during static fecal fermentations include (−)-epicatechin, (+)-catechin, 3-hydroxyphenylacetic acid (HPA), 3,4-dihydroxyphenylacetic acid (34PA), 3-(3,4-dihydroxyphenyl)propionic acid (34PP), 3-(3-hydroxyphenyl)propionic acid (HPP), 3,4-dihydroxybenzoic acid (34BA), 3-hydroxybenzoic acid (HBA), and 5-(3′,4′-dihydroxyphenyl)-*g*-valerolactone (34PV).^3^ Additionally, Stoupi et al.^9^ uniquely identified metabolites derived from procyanidin B2 and (−)-epicatechin that were larger than (−)-epicatechin (*m*/*z* > 289) and coined them as “dimeric intermediates”. However, there have been disagreements in the literature about whether several metabolites are exclusively derived from B-type, A-type, or PACs with a DP > 2.^10−14^

Ulcerative colitis (UC) is a form of inflammatory bowel disease characterized by inflammation and ulcerations in the distal colon.^15^ A consequence of UC is bacterial dysbiosis within the colon or a microbiota composition that deviates from that of healthy individuals. Common trends in bacterial dysbiosis of those with UC have been observed, and this includes decreased numbers of Bifidobacterium and Lactobacillus species, decreased alpha-diversity, increased Proteobacteria, and the presence of adherent-invasive *E. coli*.^15^ Recently, we have shown that dysbiosis can negatively impact the metabolism of PACs by decreasing the concentrations of PAC metabolites produced by UC microbiomes *ex vivo*.^16^ However, we did not elucidate in our previous work how the structural diversity of cranberry PACs may have contributed to this differential metabolism.

Although several studies have identified novel metabolites of PACs and have outlined a tentative pathway of their metabolism, there are still discrepancies in the literature over the metabolic fate of PACs.^10−14,17^ Few studies have identified changes in concentrations of precursor PACs and their dimeric intermediate metabolites throughout their metabolism.^9,10,13,17^ Furthermore, studies describing the metabolism of A-type PACs and PACs with a DP > 2 are limited.^9,10,17^ Therefore, the microbiome metabolism of cranberry A-type and B-type PACs was compared between healthy and UC microbiomes to elucidate which PACs contributed to the differential phenolic acid metabolite production demonstrated in our previous work.^16^ Additional fecal fermentations were then carried out with authentic standards of cranberry PACs that were metabolized differently between healthy and UC microbiomes to identify their derived phenolic acid metabolites.

## Materials and Methods

### Chemicals

Chromatography standards of (−)-epicatechin, procyanidin B2, 3-hydroxyphenylacetic acid (HPA), 3,4-dihydroxyphenylacetic acid (34PA), and 3-(3,4-dihydroxyphenyl)propionic acid (34PP) were acquired from Sigma-Aldrich (St. Louis, MO). Standards of 3-(3-hydroxyphenyl)propionic acid (HPP), 3,4-dihydroxybenzoic acid (34BA), and 3-hydroxybenzoic acid (HBA) were acquired from Alfa Aesar (Tewksbury, MA). 5-(3′,4′-Dihydroxyphenyl)-*g*-valerolactone (34PV) was acquired from Toronto Chemicals (North York, ON, Canada). Procyanidin A2 was purchased from Extrasynthese (Lyon, France). LC-MS-grade mobile phases were also from Sigma-Aldrich, and formic acid was from Fisher Scientific (Hampton, NH).

### Preparation of a High-PAC Cranberry Extract

A cranberry PAC extract was prepared from a high-PAC powder provided by Ocean Spray Cranberries, Inc. (Middleborough, MA). Cranberry powder (0.5 g) was dissolved in 50 mL of 0.01% (v/v) formic acid and partitioned from a 10 g C18 solid-phase extraction column (35 cm^3^, 55–105 μm; Waters, Milford, MA) first conditioned with 1 column volume (CV) of 100% methanol and 1.5 CV of 0.01% (v/v) formic acid. Cranberry polyphenols were washed with 1 CV of 0.01% (v/v) formic acid and eluted with 0.01% (v/v) formic acid in methanol. Residual methanol was then evaporated under reduced pressure, and cranberry polyphenols were dissolved in 0.01% (v/v) formic acid. Total soluble polyphenols was determined by the Folin–Ciocalteu assay, and total PACs were determined by the 4-dimethylaminocinnamaldehyde (DMAC) assay.^18,19^

### Recruitment of Subjects

Clinical trial protocols were approved by the Texas A&M University Institutional Review Board (TAMU IRB# 2017-0568D). Healthy subjects (*n* = 5) qualified if they were 18–65 years of age and had no history of chronic diseases. Subjects were excluded from the healthy group if they had a history of alcohol or substance abuse, had recurrent hospitalizations, have had seizures, taken antibiotics in the last 6 months, were lactose-intolerant and gluten-sensitive/had celiac disease, smoked more than one pack of cigarettes a week, and had renal or liver dysfunction and if females were currently pregnant or lactating. The same inclusion and exclusion criteria applied to UC subjects (*n* = 4); however, these subjects additionally reported the severity of their disease as diagnosed by a physician.

### *Ex Vivo* Fermentation of a High-PAC Cranberry Extract by Healthy and UC Microbiomes

The cranberry extract, adjusted to 600 mg L^–1^ total polyphenols, was fermented with fecal microorganisms from each of the nine donors separately to simulate microbiome metabolism by healthy and UC donors of PACs according to the procedure outlined by Sirven et al.^16^ A control fermentation containing no added polyphenols was prepared to account for any residual dietary polyphenols present and any metabolites that could form from the subject’s stool alone. Fermentations were prepared in triplicate for each time point at 0, 6, 12, 24, and 48 h. At each designated time, aliquots were acidified with formic acid and stored at −80 °C before quantifying changes in the concentration of PACs and phenolic acid metabolites via LC-ESI-IT-MS/MS and LC-ESI-QqQ-MS/MS.

### Preparation of Fermentation Aliquots for LC-ESI-IT-MS/MS and LC-ESI-QqQ-MS/MS Analysis

Acidified aliquots (250 μL) from each time point in the fermentation were mixed with 50 μL of 25 mg L^–1^ ethyl gallate in water as an internal standard. The solution was then diluted with 300 μL of 0.1% formic acid in water. A 200 mg C18 column (Waters, Milford, MA) was conditioned with 1 mL of methanol followed by 1.5 mL of 0.1% formic acid in water before the fermentation aliquot solution was loaded onto the column. The cartridge was washed with 1.5 mL of 0.1% formic acid in water and eluted with 750 μL of 0.1% formic acid in methanol. Each fermentation aliquot was extracted in triplicate at each time point. To control for matrix effects, standard solutions were prepared in the control fermentation extracted under identical conditions, except that the internal standard was replaced with 0.1% formic acid in water.

### LC-ESI-IT-MS/MS Untargeted Identification and Quantification of PACs and Identification of PAC Metabolites

Cranberry PACs were analyzed via a Thermo Finnigan LCQ Deca LC-MS instrument equipped with an electrospray ionization (ESI) source and an ion trap as the mass analyzer. In addition, a photodiode array (PDA) detector was utilized to monitor changes in the PACs and metabolites at 280 nm. Compounds were separated on a Kinetex C18 column (2.6 μM, 4.6 × 150 mm^2^) with 0.1% formic acid as mobile phase A and 0.1% formic acid in methanol as mobile phase B. The flow rate was 450 μL min^–1^ and gradient elution began with 90% A and 10% B for 5 min, decreased to 70% A after 40 min, decreased to 10% A after 45 min, and increased to 90% after 50 min, and the column was equilibrated for an additional 5 min with isocratic conditions before injection of another sample. MS was run in the negative ionization mode with a capillary temperature of 320 °C, source temperature of 350 °C, and sheath gas and auxiliary gas of 30 and 10 arbitrary units, respectively. Source parameters and collision energy were optimized by utilizing an A-type PAC trimer present in the cranberry extract. The mass range was set from *m*/*z* 150–1500. PACs were quantified in (−)-epicatechin equivalents (*R*^2^ = 0.9979, *Y* = 1.20^8^*X* + 6.09^6^).^20^

### *Ex Vivo* Fermentation of Procyanidins A2 and B2

Simulated microbiome metabolisms of procyanidin A2 and B2 standards were characterized utilizing the procedure by Sirven et al.^16^ with some modifications. A stool sample was collected from a recruited healthy subject (*n* = 1) and within 2 h of defecation was further processed inside an anaerobic chamber. The chamber (Coy Laboratory Products, Grass Lake, MI) was held at 37 °C and regulated with nitrogen, hydrogen (5%), and carbon dioxide (5%). Resazurin strips (Sigma-Aldrich, St. Louis, MO) were utilized to confirm anaerobic conditions inside the chamber during fermentations. A fecal slurry was produced by mixing 5 g of feces with 50 mL of prereduced and sterile, pH 7.5 phosphate buffered saline with added sodium thioglycolate and l-cysteine (Anaerobe Systems, Morgan Hill, CA). 481.83 μM procyanidin A2 or 480.15 μM B2 (5 mg each) was solubilized in 18 mL of fecal fermentation media as described by Tzounis et al.^21^ that contained 2.0 g of peptone water, 0.5 g of bile salts, 2.0 g of yeast extract, 0.5 g of l-cysteine, 0.05 g of hemin, 0.01 mL of vitamin K, 0.001 g of resazurin, 0.01 g of CaCl_2_·6H_2_0, 0.01 g of MgSO_4_·7H_2_0, 0.04 g of KH_2_PO_4_, 0.04 g of K_2_HPO_4_, 0.10 g of NaCl, 2.0 g of NaHCO_3_, and 2.0 mL of Tween 80 per 1 L supplied by Anaerobe Systems (Morgan Hill, CA). To simulate microbiome metabolism of procyanidin dimers, 2 mL of fecal slurry was added to fermentation vessels, while 18 mL of media and 2 mL of fecal slurry were prepared as a control. Slurries were fermented for 48 h, and 1 mL aliquots were taken after 0, 6, 12, 24, and 48 h and acidified with 5 μL of 85% formic acid. All aliquots were stored at −80 °C until analysis of fecal metabolites and parent compounds via LC-ESI-IT-MS/MS or LC-ESI-QqQ-MS/MS.

### LC-ESI-QqQ-MS/MS Quantification of PAC Metabolites 34PV, 34PP, HPP, 34PA, HPA, 34BA, and HBA

Quantification of procyanidin A2 and B2 metabolites produced during fecal fermentation was analyzed with the method outlined by Sirven et al.^16^ Briefly, metabolites were analyzed utilizing an Ultimate3000 UPLC equipped with a Thermo Scientific TSQ Quantiva triple quadrupole mass spectrometer with an ESI source. A Synergi Fusion RP column (150 × 2 mm^2^, 4 μm) was utilized with 0.1% (v/v) formic acid as mobile phase A and 0.1% (v/v) formic acid in methanol as mobile phase B. The flow rate was 400 μL min^–1^ and gradient elution began with 10% B, increased to 40% B after 5 min, and then increased to 95% B after 7 min. After 8 min, the column was re-equilibrated with 10% B until 13 min elapsed. MS data were acquired in negative polarity, and parameters consisted of 2300 V spray voltage, a sheath gas of 50, an auxiliary gas of 15, a sweep gas of 1, ion transfer tube temperature set at 350 °C, and vaporizer temperature set at 400 °C. Scheduled multiple reaction monitoring (MRM) was used to identify and quantify the metabolites based on optimized conditions and transitions of their respective standards. Additional transitions were utilized as qualifying ions for each compound. All compounds were quantified utilizing a standard curve prepared with authentic standards in the matrix. All qualifying ions, optimized collision energy and RF lens, calibration curve equations, *R*^2^, limit of detection (LOD), and limit of quantification (LOQ) information can be found in Table 1.

**Table 1:** LC-ESI-QqQ-MS/MS Conditions and Quantification Details of PAC Metabolites

compound	RT^a^	precursor (*m*/*z*)	qualifying ions^b^	collision energy	RF lens	calibration equation	*R*^2^	LOD	LOQ
34PP	5.16	181	109	17.1	75	*Y* = 3.64 × 10^5^*X* + 1.28 × 10^5^	0.9959	0.95	2.88
			135	16.9					
			137*	10.2					
HPP	6.80	165.2	106.1	20.7	40	*Y* = 5.22 × 10^5^*X* + 1.91 × 10^6^	0.9939	6.84	20.73
			119.1	14.2					
			121.2*	10.6					
34PA	3.93	167	93	21.4	30	*Y* = 7.51 × 10^4^*X* + 3.08 × 10^5^	0.9926	12.26	37.16
			95	18.8					
			123.1*	10.2					
HPA	5.67	151.1	65	24.1	30	*Y* = 2.63 × 10^4^*X* + 3.78 × 10^4^	0.9959	6.35	19.24
			79	21.2					
			92	25.8					
			107*	10.2					
34PV	5.40	207	121.9	20.6	52	*Y* = 2.63 × 10^6^*X* + 6.44 × 10^5^	0.9936	0.73	2.21
			161	20.9					
			163.1*	16.1					
34BA	3.95	153	81.2	22.7	30	*Y* = 2.18 × 10^6^*X* + 2.38 × 10^4^	0.9965	0.67	2.04
			91.1	26.9					
			109.1*	13.6					
HBA	5.90	137.1	65.1	23.7	46	*Y* = 3.05 × 10^5^*X* + 3.53 × 10^4^	0.9967	0.71	2.16
			93*	10.2					
			136.5	10.2					
ethyl gallate	5.20	197.1	124*	22.5	62	*Y* = 3.33 × 10^6^*X* + 1.83 × 10^6^	0.9927	1.34	4.07
			125	20.5					
			169	15.5					

a Retention time.

b The quantifying ion is indicated by an asterisk (*).

### Statistics

Cranberry PAC metabolism was compared between healthy and UC microbiomes at each time point of the fermentation using the Mann–Whitney *U* test with significance considered at *p* < 0.05, unless otherwise stated. Metabolites of procyanidin A2 and B2 were compared at each time point utilizing the Welch two-sample *t* test with significance considered at *p* < 0.05.

## Results and Discussion

### Comparison of the Metabolism of Cranberry PACs between Healthy and UC Microbiomes

**Figure 1:**
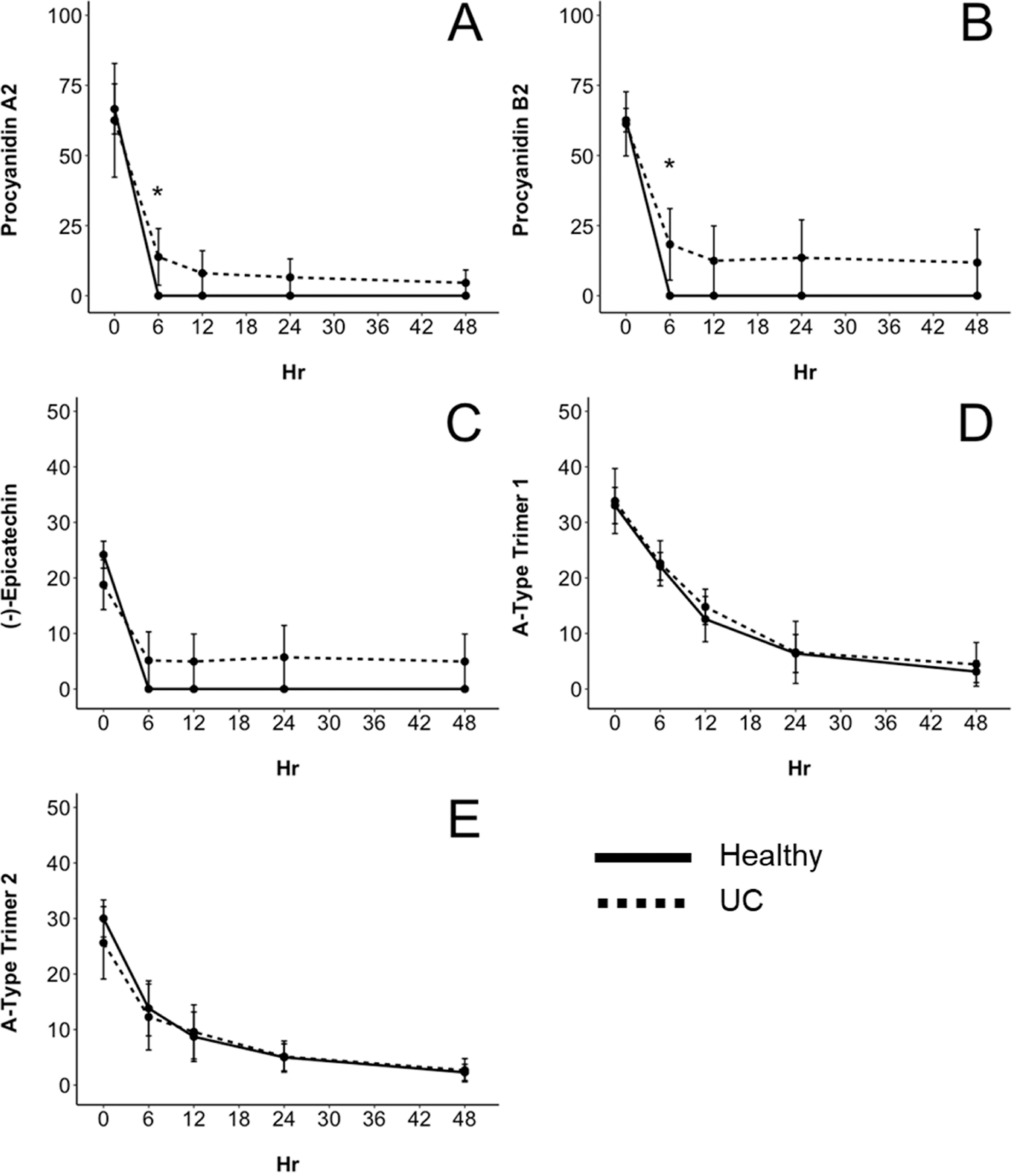
Concentration (μM) of cranberry PACs during the 48 h fermentation period quantified via LC-ESI-IT-MS/MS in (−)-epicatechin equivalents. Data are reported as mean ± SEM. (*) indicates differences (*p* < 0.10) between concentrations of PACs in either healthy (solid line) or ulcerative colitis (dashed line) fecal fermentations. Compounds identified and quantified include procyanidin A2 (A), procyanidin B2 (B), (−)-epicatechin (C), A-type PAC trimer 1 (D), and A-type PAC trimer 2 (E).

A high-PAC cranberry extract was fermented with human fecal microorganisms from either healthy or UC donors to elucidate how bacterial dysbiosis may alter the metabolism of PACs with differing DP and interflavan bond types. Identification and quantification of each PAC in the cranberry extract utilizing LC-ESI-IT-MS/MS are presented in Table 2. The concentration of total PACs in the extract before dilution in the fecal fermentation vessel was 12,674.24 ± 511.41 μM, which was 37.55 ± 2.13% of the total amount of polyphenols and higher than any other type of polyphenol contained in the extract.^16^ The cranberry extract contained 78.77 ± 7.89 μM procyanidin A2 and 62.00 ± 5.17 μM procyanidin B2 at hour 0. After 6 h of fermentation with fecal microorganisms from healthy individuals, procyanidin A2 and B2 in the cranberry extract could no longer be detected, indicating that the healthy group’s microbiota completely metabolized both dimers (Figure 1A,B). By contrast, procyanidin A2 and B2 were still present after 48 h of fermentation with fecal microorganisms from individuals with UC, with 4.58 ± 3.05 μM (5.81% remaining) and 11.81 ± 7.87 μM (19.04% remaining), respectively. Additionally, (−)-epicatechin (21.78 ± 2.43 μM) present in the cranberry extract at 0 h was not detected in the healthy group after 6 h but was still present in the UC group with 4.94 ± 4.94 μM (22.68% remaining) after 48 h (Figure 1C).

**Table 2:** LC-ESI-IT-MS/MS Identification and Characterization of Proanthocyanidins (PACs) in the Cranberry Extract Fermented with Fecal Microorganisms from Healthy and UC Fecal Donors

proanthocyanidin	concentration (μM) mean + SEM	degree of polymerization	[M – H]^−^	MS^2^
(−)-epicatechin	21.78 ± 2.43	1	289	245
procyanidin A2	78.77 ± 7.89	2	575	449, 423, 289
procyanidin B2	62.00 ± 5.17	2	577	451, 425, 407, 289
A-type PAC trimer 1 (Epi-A-Epi-Epi)	33.40 ± 2.75	3	863	711, 573, 451, 411, 289
A-type PAC trimer 2 (Epi-Epi-A-Epi)	28.05 ± 3.28	3	863	711, 575

There were two A-type trimers identified in the cranberry extract (Table 2). A-type trimer 1 was characterized by a precursor ion of *m*/*z* 863 and MS^2^ fragments of *m*/*z* 711 and 573, indicating that the A-type bond is between the extension and middle unit.^22^ A-type trimer 2 was characterized also by a precursor ion of *m*/*z* 863 but had MS^2^ fragment ions of *m*/*z* 711 and 575, indicating that the A-type bond is between the terminal and middle units.^22^ There were no differences (*p* < 0.05) in the metabolism of A-type PAC trimers between healthy and UC microbiomes (Figure 1D,E). After 24 h, ∼80% of both trimers were metabolized for both the healthy and UC groups, in comparison to the dimers which were both completely metabolized by the healthy group within 6 h. This is in accordance with Engemann et al.,^17^ who found that about 40% of an A-type PAC trimer was metabolized in comparison to 80% of an A-type PAC dimer after fermentation with pig cecum microorganisms. In our previous work, we demonstrated that microbiome dysbiosis as a consequence of UC resulted in significantly lower (*p* < 0.05) production of the PAC metabolites 34PV and HPA.^16^ To the best of our knowledge, 34PV has not been identified as a metabolite of A-type trimers but has been identified as a metabolite of procyanidin B2, (−)-epicatechin, and recently procyanidin A1 isolated from peanuts.^3,10,11^ However, HPA has been identified as a metabolite of the A-type trimer cinnamtannin B1 isolated from litchi.^17^ Considering that there were no differences in the extent of metabolism of PAC trimers between UC and healthy microbiomes and there were differences in the metabolism of both procyanidin A2 and B2, it is unlikely that the significantly higher concentrations of phenolic acid metabolites produced by healthy microbiomes in comparison to UC microbiomes that occurred in our previous work were a result of PAC trimer metabolism.

**Figure 2:**
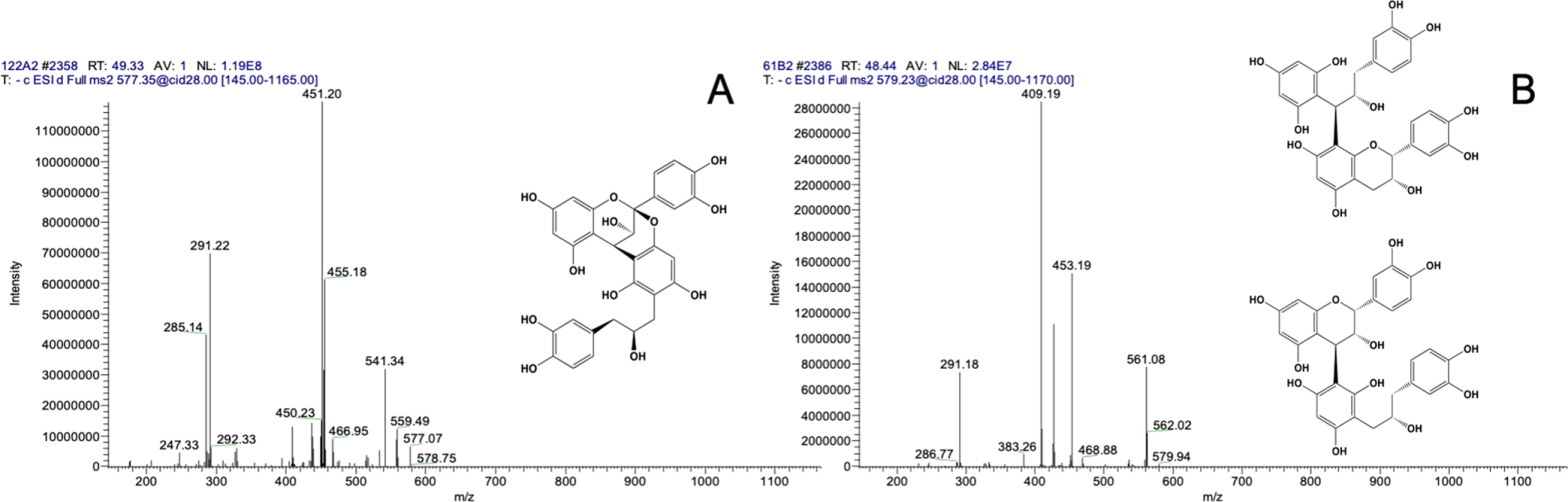
Identification of microbial-derived dimeric metabolites via LC-ESI-IT-MS/MS. The MS^2^ spectrum of these microbial metabolites derived from procyanidin A2 (A) and procyanidin B2 (B) is presented where *m*/*z* 291 indicates C-ring fission within one of the (−)-epicatechin monomers. Above each spectrum is the tentative structure of these compounds adapted from Engemann et al.^17^ and Stoupi et al.,^9^ where the dimeric metabolite of procyanidin B2 has two possible positions where C-ring fission could have occurred.

**Figure 3:**
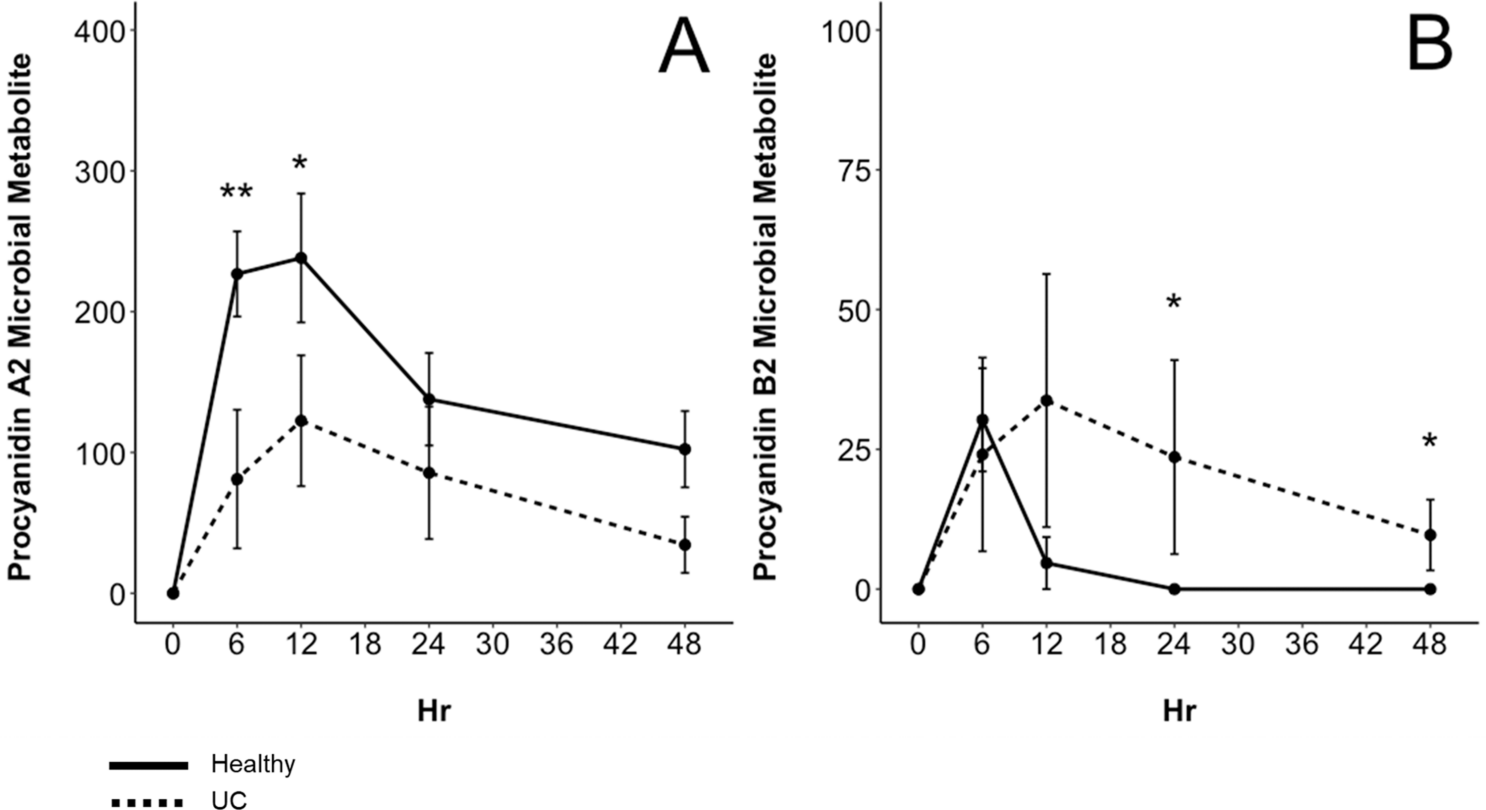
Concentration (μM) of microbial dimeric metabolites (Figure 2) identified in the cranberry extract during the 48 h fermentation period. Data are reported as mean ± SEM. (**) indicates significant differences (*p* < 0.05) between concentrations of metabolites produced from either healthy (solid line) or ulcerative colitis (dashed line) fecal donors. (*) indicates that *p* < 0.10. Procyanidin A2 dimeric microbial metabolite (*m*/*z* 577) (A) and procyanidin B2 dimeric microbial metabolite (*m*/*z* 579) (B).

Dimeric intermediate metabolites have been identified as products of PAC metabolism and may elucidate the initial differences in microbiome metabolism between procyanidin A2 and B2 dimers.^9^ Utilizing untargeted LC-ESI-IT-MS/MS analysis, two dimeric microbial metabolites were identified in this study as derivatives of procyanidins A2 and B2 (Figure 2). One of the dimeric metabolites had a precursor *m*/*z* of 577 and MS^2^ fragments of 451 and 291 (Figure 2A). This compound was previously identified after fermentation of litchi PACs by Engemann et al.^17^ as procyanidin A2 that had undergone C-ring fission in the terminal (−)-epicatechin unit as indicated by the *m*/*z* 291 fragment ion. Similar to the procyanidin A2 dimeric metabolite (*m*/*z* 577), another dimeric metabolite was identified with a precursor ion of *m*/*z* 579 that also had the fragment ion *m*/*z* 291 as a result of C-ring fission. The metabolite also had fragment ions of *m*/*z* 453 and 409, which are characteristic of PAC dimers fragmented through the retro-Diehls Alder reaction (*m*/*z* 409) and the loss of phloroglucinol (*m*/*z* 453) (Figure 2B). This dimeric metabolite was also identified by Stoupi et al.^9^ after fecal fermentation of procyanidin B2. Tentative structures are shown in Figure 2 above the corresponding mass spectra. The microbiomes of healthy individuals on average produced higher concentrations of the procyanidin A2 dimeric metabolite (*m*/*z* 577) from 6 to 48 h (Figure 3A). For both healthy and UC donor groups, this metabolite reached its maximum concentration after 12 h with 238.11 ± 45.75 μM for the healthy group and 122.53 ± 46.44 μM for the UC group. The concentration of this metabolite decreased after 12 h but was still present after 48 h in both healthy and UC fermentations with 102.32 ± 27.08 and 34.42 ± 19.95 μM, respectively. By contrast, the procyanidin B2 dimeric metabolite (*m*/*z* 579) reached its maximum concentration of 30.28 ± 9.23 μM in the healthy group after 6 h and was not detected in the healthy group again after 12 h (Figure 3B). However, the procyanidin B2 dimeric metabolite reached its maximum concentration in the UC group at 33.73 ± 22.63 μM after 12 h and was still detected and higher (*p* = 0.066) after 48 h with 9.67 ± 6.33 μM. This demonstrates that procyanidin B2 and procyanidin A2 are more extensively metabolized by healthy microbiomes compared to dysbiotic microbiomes. In addition, the significant difference in the production of dimeric metabolites between healthy and UC microbiomes may explain the difference in downstream phenolic acid metabolite production from the cranberry PAC extract that was shown in our previous work.^16^

### Comparison of the Metabolism of Procyanidins A2 and B2 by Fecal Microorganisms

**Figure 4:**
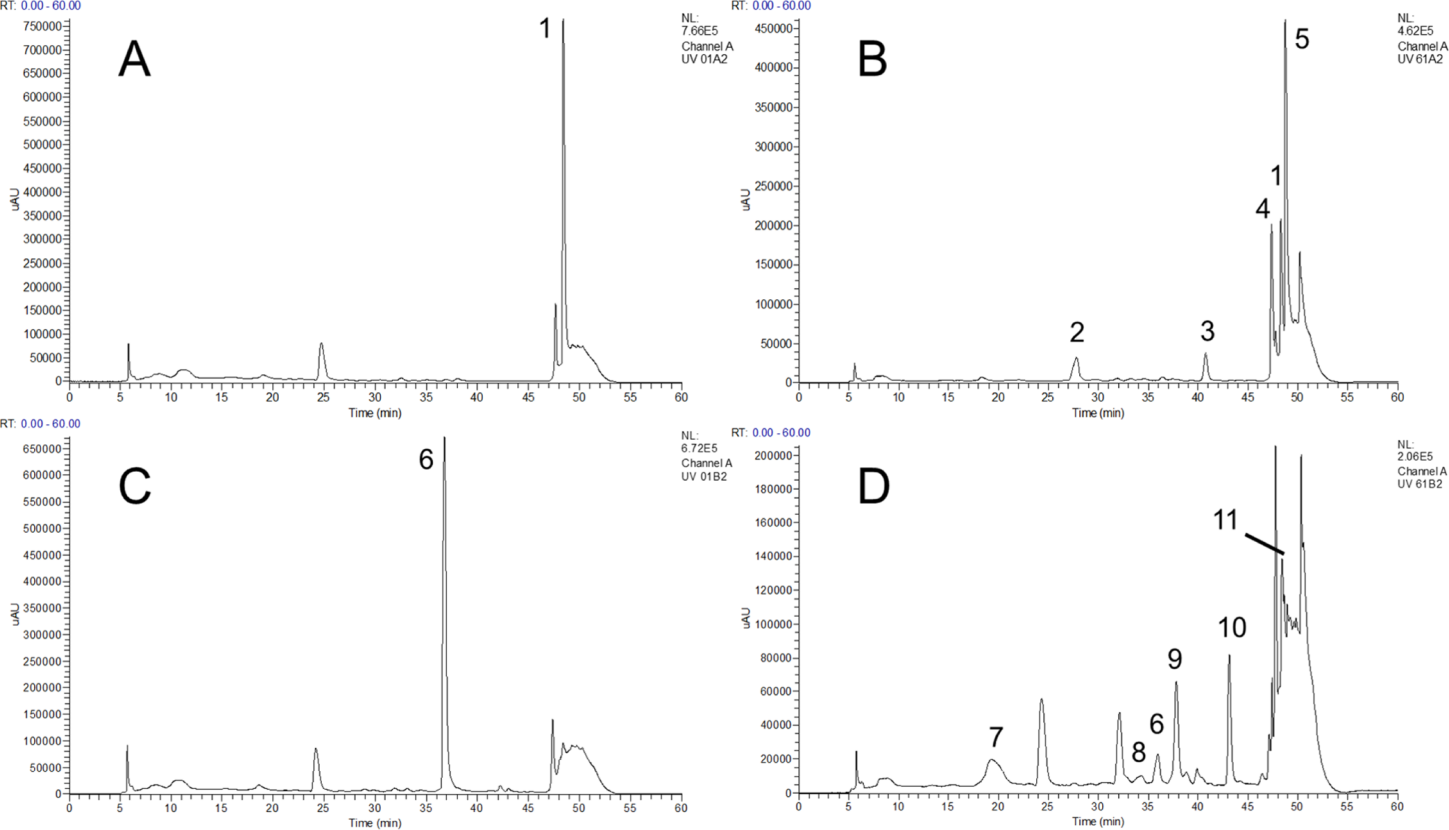
Comparison of the metabolism of procyanidin A2 (A, B) and procyanidin B2 (C, D) after 0 (A, C) and 6 h (B, D) of fermentation with fecal microorganisms from a healthy individual at 280 nm. Peak identifications include (1) procyanidin A2, (2) procyanidin A2 epimer 1, (3) procyanidin A2 epimer 2, (4) procyanidin A2 epimer 3, (5) procyanidin A2 dimeric microbial metabolite, (6) procyanidin B2, (7) (+)-catechin, (8) procyanidin B2 epimer, (9) 5-(3′,4′-dihydroxyphenyl)-*g*-valerolactone (34PV), (10) 3,4-dihydroxyphenyl-trihydroxy phenyl propan-2-ol, and (11) procyanidin B2 dimeric microbial metabolite.

**Figure 5:**
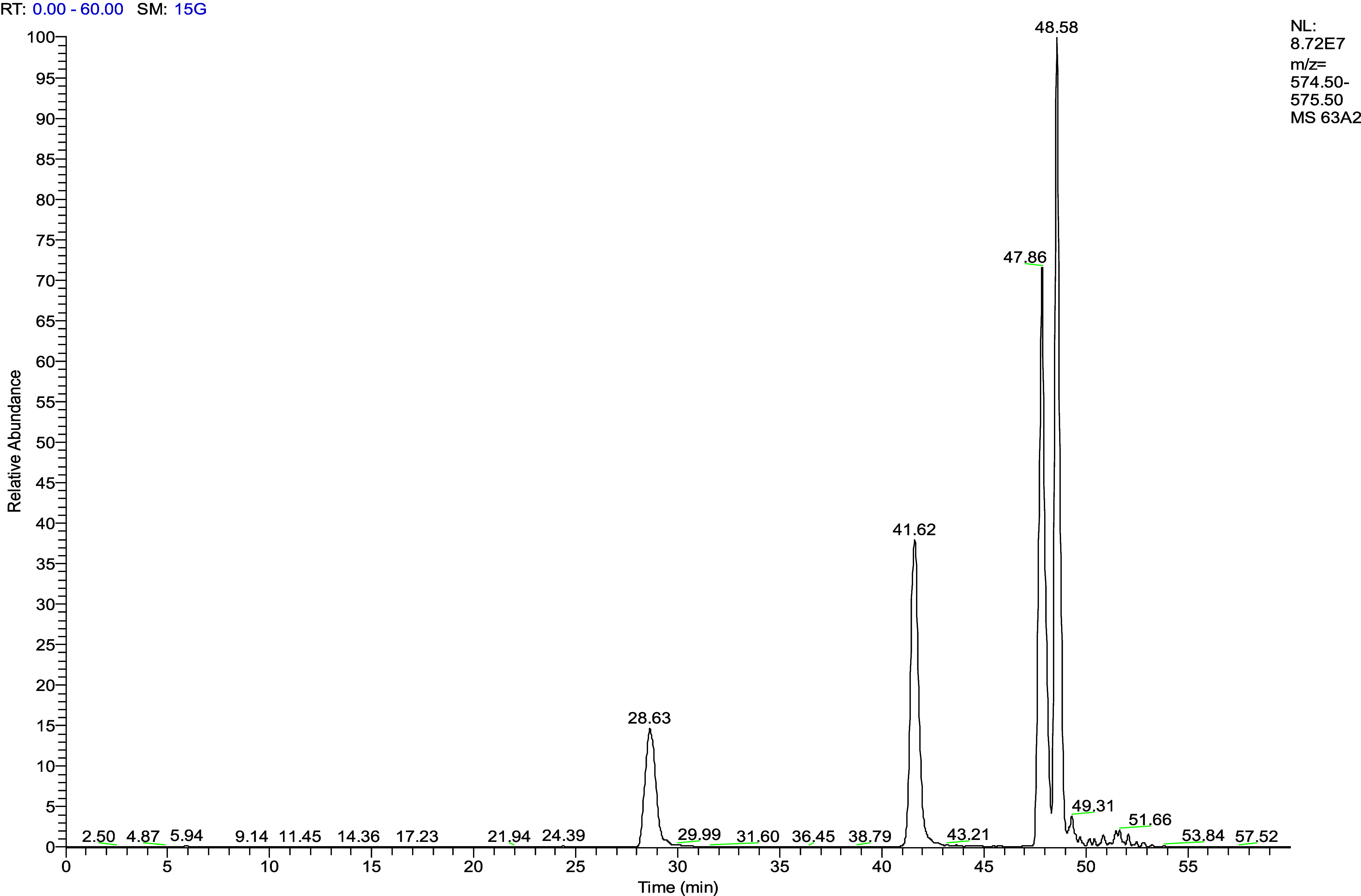
Extracted ion chromatogram of compounds that have an ion of *m*/*z* 575 after 6 h of fermentation of procyanidin A2 with fecal microorganisms from a healthy individual. Peaks with retention times of 28.63, 41.62, and 47.86 min are identified as epimers of procyanidin A2 (48.58 min).

**Figure 6:**
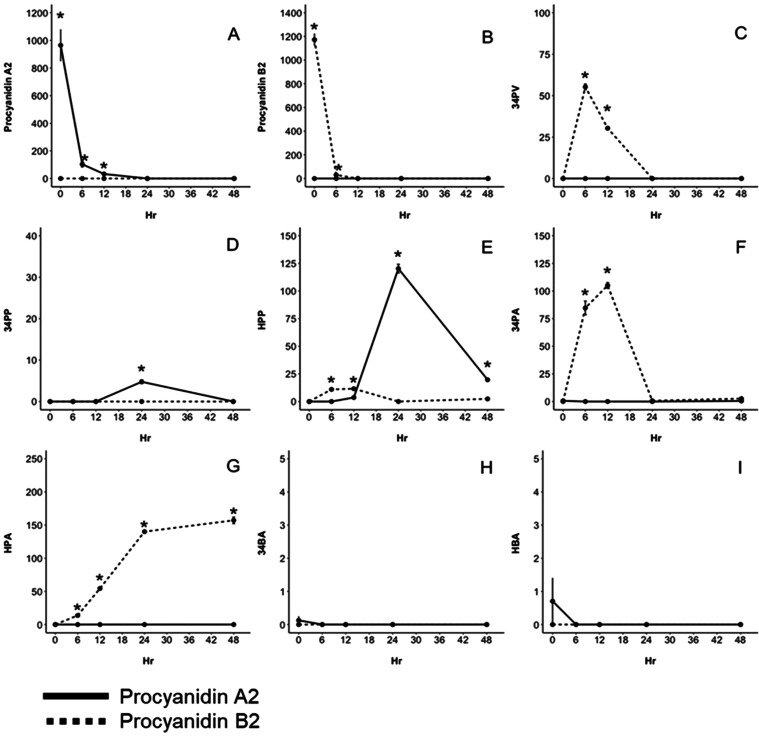
Concentration (μM) of microbial metabolites produced from procyanidin A2 (solid line) and procyanidin B2 (dashed line) after 48 h of fermentation with fecal microorganisms from a healthy donor. Data are reported as mean ± SEM (*) indicate significant differences (*p* < 0.05) between concentrations of metabolites produced from procyanidin A2 or procyanidin B2 at each hour. Compounds identified include procyanidin A2 (A), procyanidin B2 (B), 5-(3′,4′-dihydroxyphenyl)-*g*-valerolactone (34PV) (C), 3-(3,4-dihydroxyphenyl) propionic acid (34PP) (D), 3-(3-hydroxyphenyl) propionic acid (HPP) (E), 3,4-dihydroxyphenylacetic acid (34PA) (F), 3-hydroxyphenylacetic acid (HPA) (G), 3,4-hydroxybenzoic acid (34BA) (H), and hydroxybenzoic acid (HBA) (I).

Procyanidins A2 and B2 are both PAC dimers of (−)-epicatechin found in cranberries; however, procyanidin A2 differs structurally from procyanidin B2 by an additional ether bond between the two (−)-epicatechin monomers. It was found here that healthy microbiomes metabolized procyanidins A2 and B2 to a greater extent than UC microbiomes. In addition, UC microbiomes produced significantly different concentrations of dimeric microbial metabolites (Figures 1 and 3). Therefore, the intestinal metabolism of procyanidins A2 and B2 was compared *ex vivo* to identify which targeted phenolic acid metabolites are produced from each dimer. There were significant differences in the concentrations and diversity of microbial-derived phenolic acid metabolites and nonmicrobial-derived compounds produced between procyanidins A2 and B2. After 6 h, fermentation of procyanidin B2 resulted in the identification of five fecal microbial metabolites including (+)-catechin, 34PA, 34PV, 3,4-dihydroxyphenyl-trihydroxy phenyl propan-2-ol, and a procyanidin B2 dimeric metabolite (*m*/*z* 579) compared to only one metabolite produced from procyanidin A2 (Figure 4 and Table 3). Procyanidin A2 epimerized into three epimers (*m*/*z* 575) after 6 h that were still detectable after 48 h (Figures 4 and 5). By contrast, only one procyanidin B2 epimer was identified during the fermentation period. In a rat fecal fermentation model by Chen et al.,^10^ it was found that procyanidin A1 also epimerized into three isomers in both a nonmicrobial-containing control and the fermentation group. Furthermore, it was previously reported that at physiological pH, PACs can undergo conformational changes into epimers of the same polymer size.^23^ Therefore, the development of procyanidin A2 epimers was a likely consequence of the low-acid conditions of the microbiome model. This effect likely contributed to the ∼90% decrease in the procyanidin A2 concentration after 6 h, like that of procyanidin B2, yet resulted in lower concentrations and fewer microbial-derived metabolites after 6 h (Figures 4 and 6A,B). This trend of procyanidin B2 metabolizing into a larger diversity of microbial metabolites and limited microbial metabolism of procyanidin A2 carried throughout the 48 h fermentation.

**Table 3:** LC-ESI-IT-MS/MS Identification and Characterization of Procyanidin A2 and B2 Metabolites Shown in Figure 4^a^

number	compound	[M – H]^−^	MS^2^
1	procyanidin A2	575	449, 423, 289
2	procyanidin A2 epimer 1	575	
3	procyanidin A2 epimer 2	575	
4	procyanidin A2 epimer 3	575	
5	procyanidin A2 dimeric microbial metabolite	577	451, 291
6	procyanidin B2	577	451, 425, 407, 289
7	(+)-catechin	289	245
8	procyanidin B2 epimer	577	289
9	5-(3′,4′-dihydroxyphenyl)-*g*-valerolactone (34PV)	207	163
10	3,4-dihydroxyphenyl-trihydroxy phenyl propan-2-ol	291	247
11	procyanidin B2 dimeric microbial metabolite	579	453, 409, 291

a Numbers correspond to those shown in Figure 4.

34PV is speculated to be one of the first metabolites produced in the pathway of the microbiome metabolism of flavan-3-ols from which other metabolites such as 34PP, 34PA, and 34BA are hypothesized to be derived.^24,25^ The highest concentration of 34PV was 55.26 ± 1.34 μM and was produced from the fermentation of procyanidin B2 after 6 h, which then decreased to 30.33 ± 0.81 μM after 12 h and was not detected thereafter (Figure 6C). During the fermentation of procyanidin A2, 34PV was not detected over the 48 h period. This agrees with Engemann et al.,^17^ who incubated procyanidin A2 isolated from litchi with porcine cecum microorganisms and did not identify 34PV as a metabolite. Furthermore, prior to the identification of 34PV as a metabolite of procyanidin A1 by Chen et al.,^10^ it has been suggested that 34PV is a unique metabolite to procyanidin B2 and (−)-epicatechin, where 34PV can be derived from procyanidin B2 directly or by the production of free (−)-epicatechin, supported by the identification of (−)-epicatechin after fermentation of procyanidin B2, estimated to account for ∼10% of procyanidin B2 metabolism by the microbiome.^3,11,12^ By contrast, concentrations of (−)-epicatechin during fermentation of procyanidin B2 were below levels of quantification in this study. However, in agreement with Selma et al.,^24^ the diastereomer of (−)-epicatechin, (+)-catechin, was identified as a microbial metabolite of procyanidin B2 in this study (Figure 4 and Table 3). Overall, the evidence here suggests that 34PV is a metabolite unique to procyanidin B2 and not procyanidin A2.

34PP and HPP are hypothesized to be microbial metabolites derived from 34PV and therefore also biomarkers of flavan-3-ol consumption.^3,25^ After fermentation of procyanidins A2 and B2, HPP was present after 12 h with 3.63 ± 0.08 and 11.58 ± 0.49 μM produced, respectively (Figure 6E). HPP produced from procyanidin A2 reached a maximum concentration after 24 h of 120.26 ± 2.04 μM and was significantly higher (*p* < 0.05) than HPP produced from procyanidin B2 from 24 to 48 h. It has been speculated that HPP is a result of dehydroxylation of 34PP.^3,25^ Although HPP was produced by both procyanidins A2 and B2, 34PP was only found after 24 h of fermentation of procyanidin A2 and was never detected in procyanidin B2 fermentations (Figure 6D). By contrast, Stoupi et al.^11^ detected 34PP between 24 and 48 h and HPP between 6 and 9 h of fermentation of procyanidin B2. Considering that 34PV was not produced by procyanidin A2 in our study and the limited production of 34PP by both dimers, our results suggest that HPP may be produced from alternative pathways other than dehydroxylation of 34PP and 34PV or that there is a high turnover rate of 34PP to HPP.

34PA and HPA have also been identified as metabolites derived from 34PP and HPP during fecal microbial fermentation of both procyanidin A-type and B-type dimers.^3,10,13,17,25^ The maximum concentration of 34PA was reached after 12 h of fermentation of procyanidin B2 with 105.04 ± 2.01 μM (Figure 6F) but was produced in appreciably lower (*p* < 0.05) concentrations from procyanidin A2 that reached a maximum concentration after 48 h that was <LOQ. Prior studies have hypothesized that 34PA is derived from the B-ring of the extension unit of procyanidin B2 or via alpha-oxidation of 34PP.^3,11,25^ The former mechanism is perhaps more plausible considering the lack of production of 34PP from procyanidin B2. The additional ether bond between C2 and C7 of the (−)-epicatechin units in procyanidin A2 may also prevent the production of 34PA from the B-ring. HPA is a dehydroxylated derivative of 34PA and reached a maximum concentration after fermentation of procyanidin B2 after 48 h with 157.16 ± 3.76 μM (Figure 5G). Results suggested that HPA was derived from 34PA considering that the concentrations of HPA produced from procyanidin B2 were comparable to that of 34PA and that 34PA was absent, while 34PP was prominent in procyanidin A2 fermentations.

Protocatechuic acid (34BA), 3-hydroxybenzoic acid (HBA), and benzoic acid have also previously been identified as microbial metabolites of both A-type and B-type PACs. Their production has been proposed as α- or β-oxidation derivatives of phenyl acetic acid and phenyl propionic acid derivatives, respectively.^3,17,25^ However, except for trace levels of benzoic acid detected only after 6 h of fermentation of procyanidin A2 (data not shown), fermentation of procyanidin A2 or B2 did not result in 34BA, HBA, or appreciable levels of benzoic acid (Figure 6H,I). This suggests that these compounds may not be important biomarkers of procyanidin A2 and procyanidin B2 microbiome metabolism.

**Figure 7:**
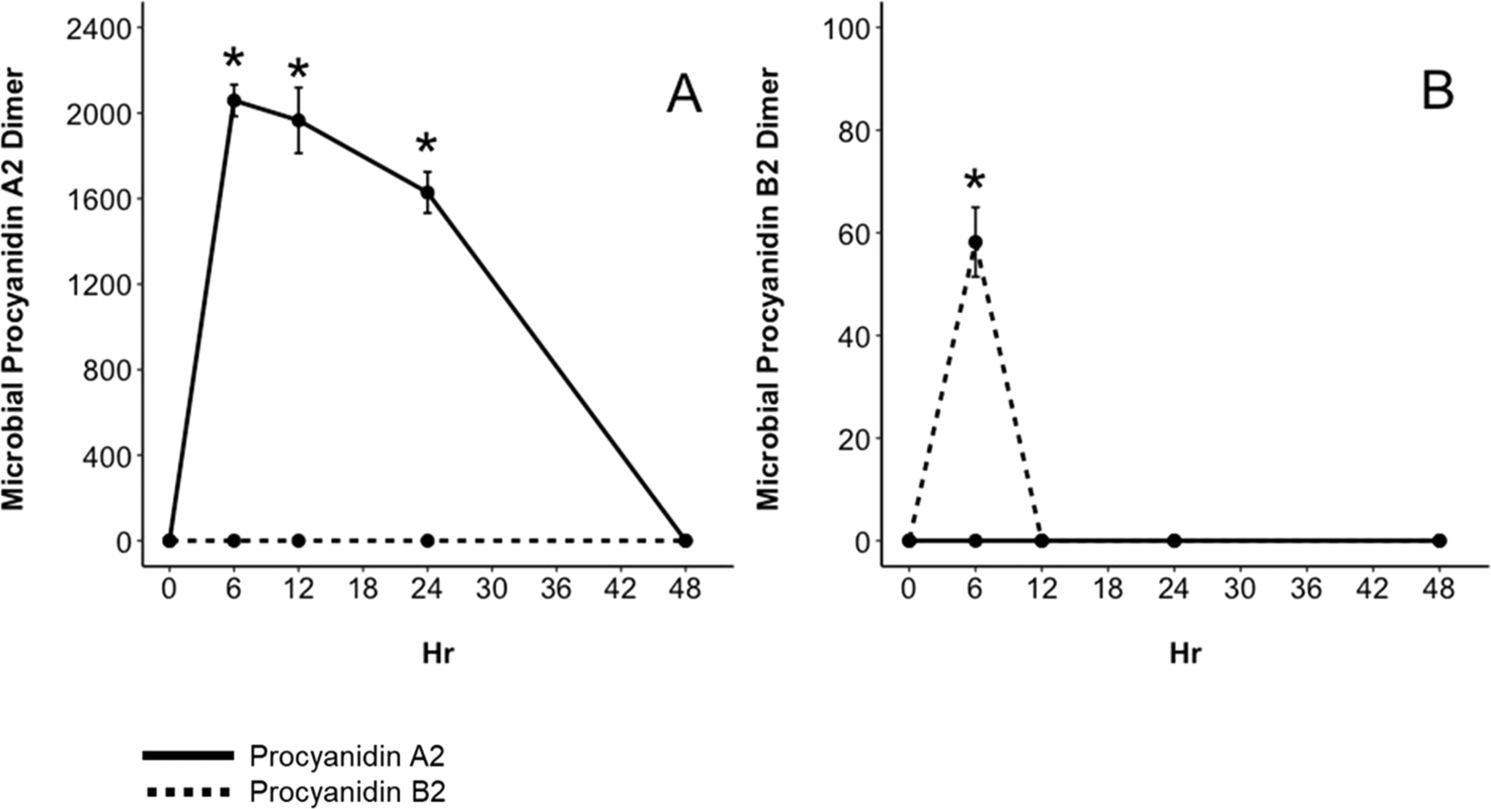
Concentration (μM) of dimeric microbial metabolites produced from procyanidin A2 (solid line) and procyanidin B2 (dashed line) after 48 h of fermentation with fecal microorganisms from a healthy donor. Data are reported as mean ± SEM. (*) indicates significant differences (*p* < 0.05) between concentrations of metabolites produced from procyanidin A2 or procyanidin B2 at each hour. Compounds identified include procyanidin A2 dimeric intermediate *m*/*z* 577 (A) and procyanidin B2 dimeric intermediate *m*/*z* 579 (B).

Dimeric metabolites were also investigated to better confirm their identity and understand the role they play in the metabolic fate of procyanidins A2 and B2. Both dimeric metabolites identified from the fecal fermentation of the cranberry extract shown in Figure 2 were also identified after fermentation of procyanidin authentic standards. The dimeric microbial metabolite of procyanidin A2 (*m*/*z* 577) reached a maximum concentration of 2059 ± 73.8 μM after 6 h and then decreased from 12 to 24 h until the compound was not detectable at 48 h (Figure 7A). This dimeric intermediate was unique to procyanidin A2 and was not detected in the fermentation of procyanidin B2. The procyanidin B2 dimeric metabolite (*m*/*z* 579) reached a maximum concentration of 58.20 ± 6.77 μM after 6 h and was not detectable thereafter (Figure 7B). In summary, procyanidin B2 was metabolized into a diversity of phenolic acid metabolites compared to procyanidin A2. These metabolites included 34PV, HPP, 34PA, and HPA with (+)-catechin, 3,4-dihydroxyphenyl-trihydroxy phenyl propan-2-ol, and a dimeric metabolite (*m*/*z* 579) as possible intermediates. Procyanidin A2 was metabolized into a dimeric microbial metabolite (*m*/*z* 577), epimerized, or formed phenyl propionic acids (34PP, HPP).

In our previous work, we showed that UC microbiomes produced significantly lower 34PV and HPA in comparison to healthy microbiomes, both of which we have found here to be unique metabolites of procyanidin B2 and not procyanidin A2. Taken together, this demonstrates that differential metabolism of PAC dimers, especially procyanidin B2, contributed to the discrepancy in phenolic acid metabolite production between healthy and UC microbiomes seen in our previous work.^16^

### Implications of the PAC DP and Interflavan Bond Type on PAC Metabolic Fate and Physiological Effects

Cranberry PACs have different routes of metabolism that can be dependent on the DP and the interflavan bond type. Procyanidin B2 was metabolized by fecal microorganisms into a variety of phenolic acid metabolites, including 34PV, HPP, 34PA, HPA, and (+)-catechin. By contrast, procyanidin A2 was metabolized into a limited number of phenolic acid metabolites, including 34PP and HPP. It has been shown *in vivo* and *in vitro* that phenolic acid metabolites may have higher bioavailability than the PACs from which they are derived.^26,27^ When comparing total molar mass recoveries of procyanidin dimers from the production of phenolic acid metabolites over 48 h, procyanidin A2 resulted in 34.47 ± 1.73% recovery, while procyanidin B2 resulted in a recovery of 154.78 ± 1.73% (data not shown). Therefore, due to the rigidity of the A-type interflavan bond, A-type PACs may result in less absorbable metabolites in comparison to B-type PACs.^3^ However, procyanidin A2 resulted in three isomers and a dimeric intermediate (*m*/*z* 577) that accumulated and resided in fermentations for a longer period of time (6–24 h) than the dimeric intermediate of procyanidin B2 (6 h). To the best of our knowledge, the absorption of dimeric intermediates of PACs has not been investigated. It has also been shown that the greater the DP or molecular weight, the lower the absorption, where several *in vivo* studies have not been able to detect PACs with a DP of 2 or greater after consumption of a high-PAC food.^26^ Therefore, procyanidin A2 and larger DP PACs more likely than procyanidin B2 may exhibit physiological effects in the gastrointestinal tract, where some authors have shown that PACs may have prebiotic effects, promote mucin production, and downregulate inflammation.^3,8^ Taken together, these findings suggest that DP and the interflavan bond type may alter the metabolic fate of PACs and therefore their physiological effects.

## Abbreviations

PAC: proanthocyanidin
DP: degree of polymerization
HPA: 3-hydroxyphenylacetic acid
34PA: 3,4-dihydroxyphenylacetic acid
34PP: 3-(3,4-dihydroxyphenyl)propionic acid
HPP: 3-(3-hydroxyphenyl)propionic acid
34BA: 3,4-dihydroxybenzoic acid
HBA: 3-hydroxybenzoic acid
34PV: 5-(3′,4′-dihydroxyphenyl)-*g*-valerolactone
UC: Ulcerative Colitis
